# Studying pulmonary fibrosis due to microbial infection *via* automated microscopic image analysis

**DOI:** 10.3389/fmicb.2023.1176339

**Published:** 2023-03-23

**Authors:** Yajie Chen, Henghui He, Licheng Luo, Kangyi Liu, Min Jiang, Shiqi Li, Xianqi Zhang, Xin Yang, Qian Liu

**Affiliations:** ^1^School of Electronic Information and Communications, Huazhong University of Science and Technology, Wuhan, China; ^2^Department of Forensic Medicine, Tongji Medical College, Huazhong University of Science and Technology, Wuhan, China

**Keywords:** microbial infection, pulmonary fibrosis, microscopic image, artificial intelligence, image analysis, macrophage

## Abstract

**Introduction:**

Pulmonary fibrosis is a consequential complication of microbial infections, which has notably been observed in SARS-CoV-2 infections in recent times. Macrophage polarization, specifically the M2-type, is a significant mechanism that induces pulmonary fibrosis, and its role in the development of Post- COVID-19 Pulmonary Fibrosis is worth investigating. While pathological examination is the gold standard for studying pulmonary fibrosis, manual review is subject to limitations. In light of this, we have constructed a novel method that utilizes artificial intelligence techniques to analyze fibro-pathological images. This method involves image registration, cropping, fibrosis degree classification, cell counting and calibration, and it has been utilized to analyze microscopic images of COVID-19 lung tissue.

**Methods:**

Our approach combines the Transformer network with ResNet for fibrosis degree classification, leading to a significant improvement over the use of ResNet or Transformer individually. Furthermore, we employ semi-supervised learning which utilize both labeled and unlabeled data to enhance the ability of the classification network in analyzing complex samples. To facilitate cell counting, we applied the Trimap method to localize target cells. To further improve the accuracy of the counting results, we utilized an effective area calibration method that better reflects the positive density of target cells.

**Results:**

The image analysis method developed in this paper allows for standardization, precision, and staging of pulmonary fibrosis. Analysis of microscopic images of COVID-19 lung tissue revealed a significant number of macrophage aggregates, among which the number of M2-type macrophages was proportional to the degree of fibrosis.

**Discussion:**

The image analysis method provids a more standardized approach and more accurate data for correlation studies on the degree of pulmonary fibrosis. This advancement can assist in the treatment and prevention of pulmonary fibrosis. And M2-type macrophage polarization is a critical mechanism that affects pulmonary fibrosis, and its specific molecular mechanism warrants further exploration.

## 1. Introduction

Microbial infections represent a major culprit behind the onset of pulmonary fibrosis. A variety of microorganisms have been identified as potential inducers of this debilitating disease, including the human T-cell leukemia virus, the human immunodeficiency virus, cytomegalovirus, Epstein–Barr virus, influenza virus, avian influenza virus, Middle East respiratory syndrome coronavirus, heavy acute respiratory syndrome coronavirus, SARS-CoV-2 (Huang and Tang, 2021), Mycobacterium tuberculosis, Chlamydia and Mycoplasma. The pathogenesis of pulmonary fibrosis due to microbial infections, especially viral infections, consists of two distinct mechanisms. The pathogenesis of viral-induced pulmonary fibrosis entails two distinct mechanisms. Firstly, direct viral damage during the infection leads to acute and heavy injury to the lungs. This acute insult results in persistent lung damage and/or abnormal wound healing, thus facilitating the progression of pulmonary fibrosis. Secondly, viral infections trigger an immune-mediated response that causes tissue damage. Upon infection, immune cells aggregate at the site of injury, releasing a vast array of pro-inflammatory and pro-fibrotic cytokines that mediate the progression of fibrosis. Consequently, the synergistic interplay between viruses and these factors culminates in sustained and substantial lung damage, ultimately leading to the development of pulmonary fibrosis.

In recent years, COVID-19 caused by SARS-CoV-2 infection has had a profound impact on the global population. In fact, over one-third of heavy COVID-19 pneumonia survivors discharged from hospitals have been found to develop pulmonary fibrosis (Han et al., 2021). Furthermore, forensic examination of deceased COVID-19 patients by the Liu Liang team has revealed copious amounts of viscous gray-white liquid and visible fibrous strands on lung sections (Liu et al., 2020). Grillo and colleagues conducted a systematic analysis of lung slice samples from eight COVID-19 patients who died in intensive care, and noted significant pulmonary fibrosis remodeling, characterized by fibroblast proliferation and alveolar occlusion (Grillo et al., 2021). This underscores the fact that pulmonary fibrosis is a significant complication that can lead to heavy illness and death in COVID-19 patients.

Tissue-resident macrophages are highly plastic cells that can polarize into classical activation phenotype (M1) macrophages or alternative activation phenotype (M2) macrophages (Sica and Mantovani, 2012; Vasse et al., 2021). M2 macrophages can be induced by various cytokines and are associated with fibrosis (Zhong et al., 2014). M2 macrophages can produce pro-fibrotic mediators such as transforming growth factor-β (TGF-β), which sustain the activation of fibroblasts and promote myofibroblast proliferation, leading to excessive deposition of extracellular matrix (ECM) and structural remodeling of lung tissue, ultimately resulting in pulmonary fibrosis and respiratory failure (Song et al., 2000). Therefore, after SARS-CoV-2 invasion, do pulmonary macrophages polarize into M2 macrophages in large numbers, participating in the initiation of pulmonary fibrosis? If the degree of lung tissue fibrosis can be correlated with the number of macrophages, it would provide solid evidence for this hypothesis.

For a long time, the gold standard for diagnosing pulmonary fibrosis has been pathological examination of the affected lung tissue, making histopathology the foundation for related research. Therefore, the analysis of microscopic images of pathological tissue sections is particularly important. However, traditional microscopic image analysis techniques have the following main limitations. Firstly, they rely on subjective judgments by humans and are experiential in nature, with results being significantly influenced by the observer’s individual biases. Secondly, image analysis and cell counting only represent partial views and are difficult to analyze globally. Finally, because different staining for different markers often requires different sections of the same tissue block, even consecutive sections on different slides are difficult to accurately match for subsequent analysis, making it challenging to perform correlation analysis of different indicators in the same area. Therefore, using artificial intelligence algorithms is of high demand to effectively solve the aforementioned problems.

This paper presents an artificial intelligence-based image analysis method for registering, cropping, and classifying fibrosis degree in lung FFPE (Formalin-Fixed and Parrffin-Embedded) slice. To automate the classification of fibrosis degree, we utilize the ResNet network (He et al., 2016) to extract high-dimensional features of lung tissue from pathological microscopy images. Then, we use the self-attention mechanism of Transformer (Dosovitskiy et al., 2020) to select the most discriminative local features. We further employ semi-supervised learning to improve classification accuracy with a large number of unlabeled pathological images. Our results demonstrate that our classification model can accurately focus on the pathological tissue in the lung and classify images in a way that mimics human interpretation. Moreover, we apply the Trimap distance method to automatically count the number of macrophages in immunohistochemistry images of lung tissue. Given the characteristics of lung tissue, such as the presence of many empty areas like blood vessels and airway cavities, we employ an effective area calibration method in addition to cell counting to better reflect the positive density of the target cells. We applied the above-mentioned intelligent analysis method for lung FFPE slice images to quantitatively analyze SARS-CoV-2-induced lung fibrosis and investigate the role of macrophage polarization in the mechanism of SARS-CoV-2-induced lung fibrosis.

## 2. Materials and methods

### 2.1. Pathological tissue samples

The data used to train the algorithm model in this paper comes from 10 lung FFPE slices of COVID-19 death cases. The experimental data comes from lung tissue samples of 4 different COVID-19 death cases with pulmonary lesions, as well as 3 lung tissue samples without pulmonary lesions. HE staining, CD68 immunohistochemical staining, and CD163 immunohistochemical staining were used as staining methods, where CD68 represents total macrophages and CD163 represents M2 macrophages. HE staining was used for the classification task, while immunohistochemical staining was used for the counting task.

### 2.2. Preparing image analysis samples *via* image registration and cropping

Due to spatial position deviation between the original HE staining images and immunohistochemical images, we registered them using affine transformation in OpenCV to achieve optimal alignment. Moreover, the original pathological image size was extremely large, ranging from 600 million to 1.2 billion pixels, which precluded direct input into the network for classification and counting. To overcome this challenge, we manually selected the region of interest and cropped it into patches with a resolution of 1,600 × 1,600 pixels, resulting in 1589 labeled patches. We separate all labeled patches into 1,082, 269, and 198 patches for training, validation, and testing, respectively. Furthermore, to enhance the classification accuracy, we obtained 1,420 unlabeled patches. We then used deep learning models to automatically classify registered HE staining patches and count macrophage on the corresponding immunohistochemical images. Finally, we spliced the target images to restore them to the original slice size and analyzed the corresponding classification and counting results in the corresponding regions.

### 2.3. Lung tissue fibrosis classification *via* deep learning

**Figure 1:**
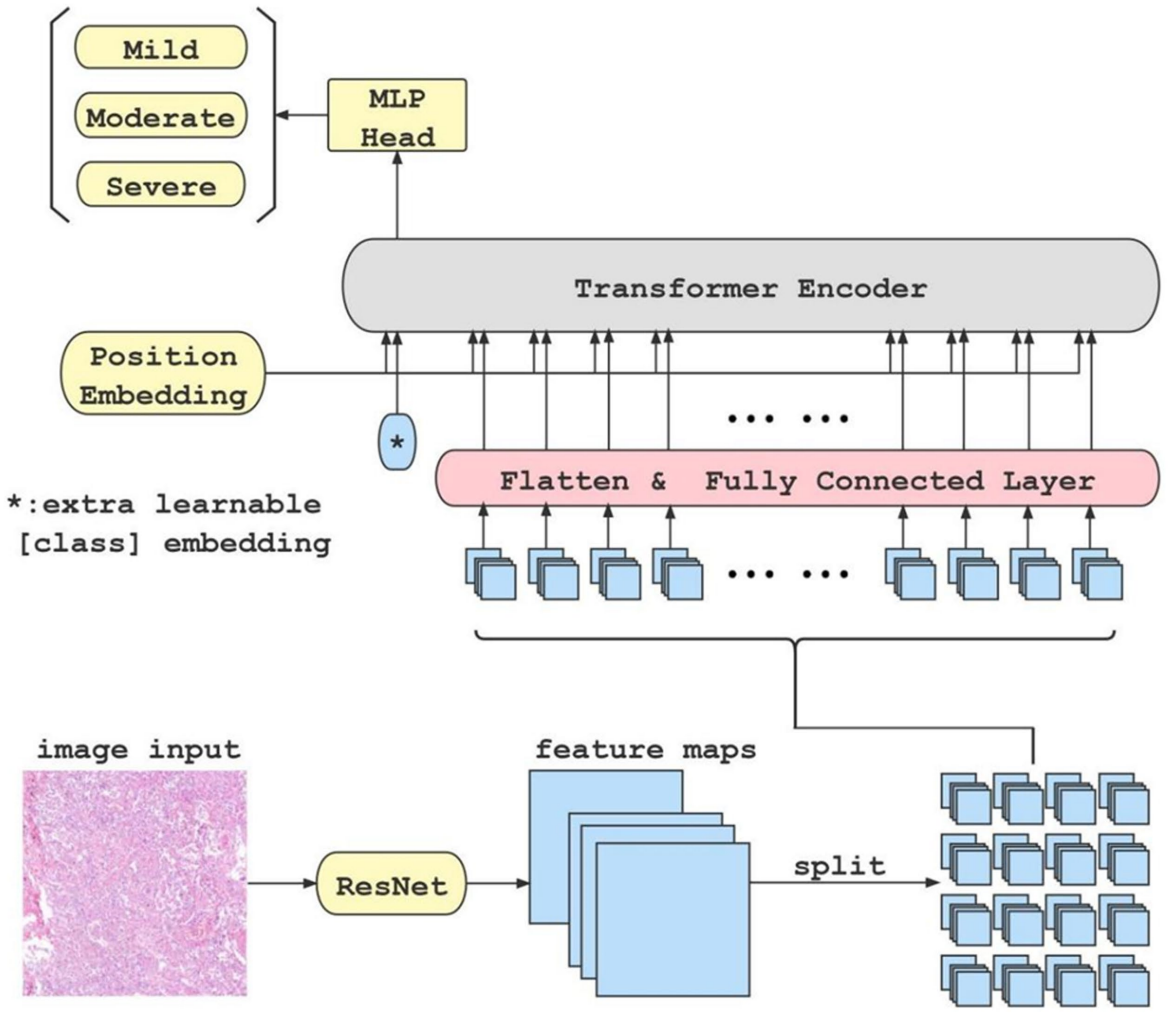
This is the flow chart of our classification model. When an original image is input to the ResNet feature extractor, the extractor outputs high-dimensional features maps of the image. Afterwards, the feature maps are split into blocks(in this case, there are 4 × 4 blocks), flattened, and fed into fully connected layer block by block. Meanwhile, a class token is initialized separately and fed into the transformer encoder together with each block of the output from the fully connected layer. Finally, the class token is extracted and fed into an MLP to get the classification result.

Various improvements have been made to the Visual Transformer (ViT) method (Dosovitskiy et al., 2020), including knowledge distillation (Touvron et al., 2021a), Re-attention (Zhou et al., 2022), and LayerScale (Touvron et al., 2021b). In this paper, we followed the automatic classification model Tokens-to-Token ViT (Yuan et al., 2021) which consists of a pre-trained ResNet backbone and a Transformer encoder-decoder. The ResNet network extracts high-dimensional features from image patches. We normalize these features and combine them with the positional encoding before they are input into the encoder-decoder with multi-head self-attention. The output of the encoder-decoder (i.e., the class token) is fed into a prediction head that is made up of fully connected layers. Then the output of the prediction head determines the specific classification (Figure 1). To further improve the classification accuracy, we employed the semi-supervised method which combines self-training and consistency learning (Tarvainen and Valpola, 2017), and designed a model from the perspective of pseudo-labeling (Arazo et al., 2020). We adopted a similar structure of Cross Pseudo Supervision (Chen et al., 2021) in our approach. The loss function is comprised of two parts: supervised and unsupervised. The supervised part calculates the cross-entropy loss between the predicted classes and the labels. For unlabeled data, one-hot pseudo-labels are generated using the output of another model, and the cross-entropy loss is calculated between the predicted values and the pseudo-labels generated by the other model. By merging the ResNet feature extractor and Transformer classifier, our approach is able to leverage both high-dimensional image features and long-term dependencies of sequence data for classification. This allows for training with advanced features, which reduces the need for extensive training data, making it ideal for medical datasets. Furthermore, through the use of semi-supervised learning, our model effectively integrates finely labeled and unlabeled data to improve classification accuracy.

### 2.4. Manual-labeled fibrosis rubric

After referencing to the refined Ashcroft score criteria (Hubner et al., 2008), our study evaluated the degree of pulmonary fibrosis in detail, assigning a score of 1–8 based on the specific morphological characteristics of the lung fibrosis. The score and detailed morphological descriptions are shown in Table 1. Based on the morphological characteristics, scores of 1–3 were classified as light, 4–5 as moderate, and 6–8 as heavy. The annotations were conducted by 5 pathologists with experience, who independently evaluated HE-stained patches in the algorithm training dataset and scored them according to the standard. The group results were then collected and compared for final calibration.

**Table 1:** Modified Ashcroft score(Hubner et al., 2008).

Rate	Description
1	Alveolar septa: Isolated gentle fibrotic changes (septum ≤3× thicker than normal)
	Lung structure: Alveoli partly enlarged and rarefied, but no fibrotic masses present
2	Alveolar septa: Clearly fibrotic changes (septum >3× thicker than normal) with knot-like formation but not connected to each other
	Lung structure: Alveoli partly enlarged and rarefied, but no fibrotic masses
3	Alveolar septa: Contiguous fibrotic walls (septum >3× thicker than normal) predominantly in whole microscopic field
	Lung structure: Alveoli partly enlarged and rarefied, but no fibrotic masses
4	Alveolar septa: Variable
	Lung structure: Single fibrotic masses (≤10% of microscopic field)
5	Alveolar septa: Variable
	Lung structure: Confluent fibrotic masses (>10% and ≤ 50% of microscopic field). Lung structure severely damaged but still preserved
6	Alveolar septa: Variable, mostly not existent
	Lung structure: Large contiguous fibrotic masses (>50% of microscopic field). Lung architecture mostly not preserved
7	Alveolar septa: Non-existent
	Lung structure: Alveoli nearly obliterated with fibrous masses but still up to several air bubbles
8	Alveolar septa: Non-existent
	Lung structure: Only fibrotic masses in microscopic field

### 2.5. Cell counting and effective area calibration

**Figure 2:**
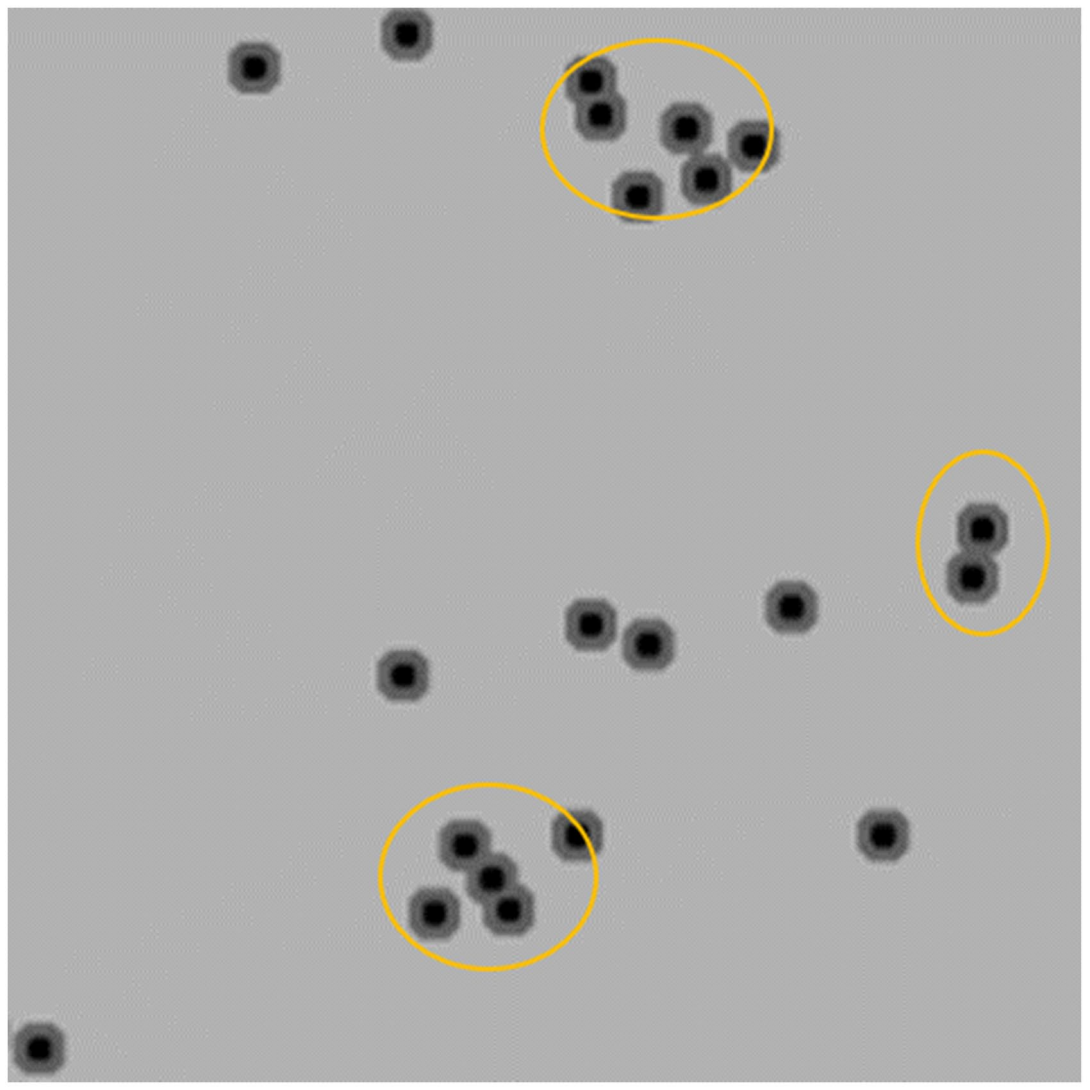
Distance map visualization.

The post-processing technique extracts the area of minimum distance in each local region of the predicted distance map, which represents the center of a cell. The number of centers detected corresponds to the number of cells in the image. Finally, the density of positive cells is calculated by adjusting for the effective tissue area. We adopt Trimap (Arteta et al., 2016) which involves three semantic segmentation classes to count the number of target cells. Specifically, the cell region is divided into two semantic classes, namely the segmentation region closer to the cell center and the cell region farther away from the center. The third semantic class is defined as the background class. To differentiate the two classes in the cell region based on proximity to the cell center, such as the orange region in Figure 2, the two regions closest to the center (black regions) can be used to separate two adherent cells. To implement Trimap, we leveraged the UNet segmentation network (Ronneberger et al., 2015) which employs a combination of upsampling and downsampling layers to extract high-level features and predict a distance map for each pixel in the input image. Since the center of a cell is typically located within regions closer to the cell centers, the post-processing algorithm extracts the local minimum region and calculates the centroid to obtain the cell center. To obtain the density of positive cells, we calculated the area of valid tissues in the patch to determine the effective tissue area. The invalid tissues are typically white in color, and threshold can be used to extract the blank connected regions in the image. Any small connected regions that are not relevant are then excluded based on their minimum area of the alveolar space in the patch. The effective tissue area is calculated by subtracting the sum of areas of all connected regions larger than the minimum area from the total area of the patch.

Finally, the density of cells in each patch is calculated as follow:


(1) $$\;density=\frac{p_{i}}{R_{e}}\;$$

(2) $$R_{e}=\frac{A_{e}}{A_{total}}\;$$

(3) $$\;\;A_{e}=A_{total}-A_{white}$$

where $R_{e}\;$ is the rate of effect area $A_{e}$ to the total area of the patch $A_{total}$ , $A_{white}$ , is the area of the white regions in the patch and $p_{i}$ is the predicted count of cells in the image.

Compared to existing counting algorithms (Wan and Chan, 2019; Wan et al., 2021), the Trimap distance algorithm is capable of handling adhesive samples by utilizing the distance between pixels and the centroid and the use of effective area calibration allows the calculation of the effective positive density in combination with the cell count.

## 3. Results

### 3.1. Image registration and cropping

**Figure 3:**
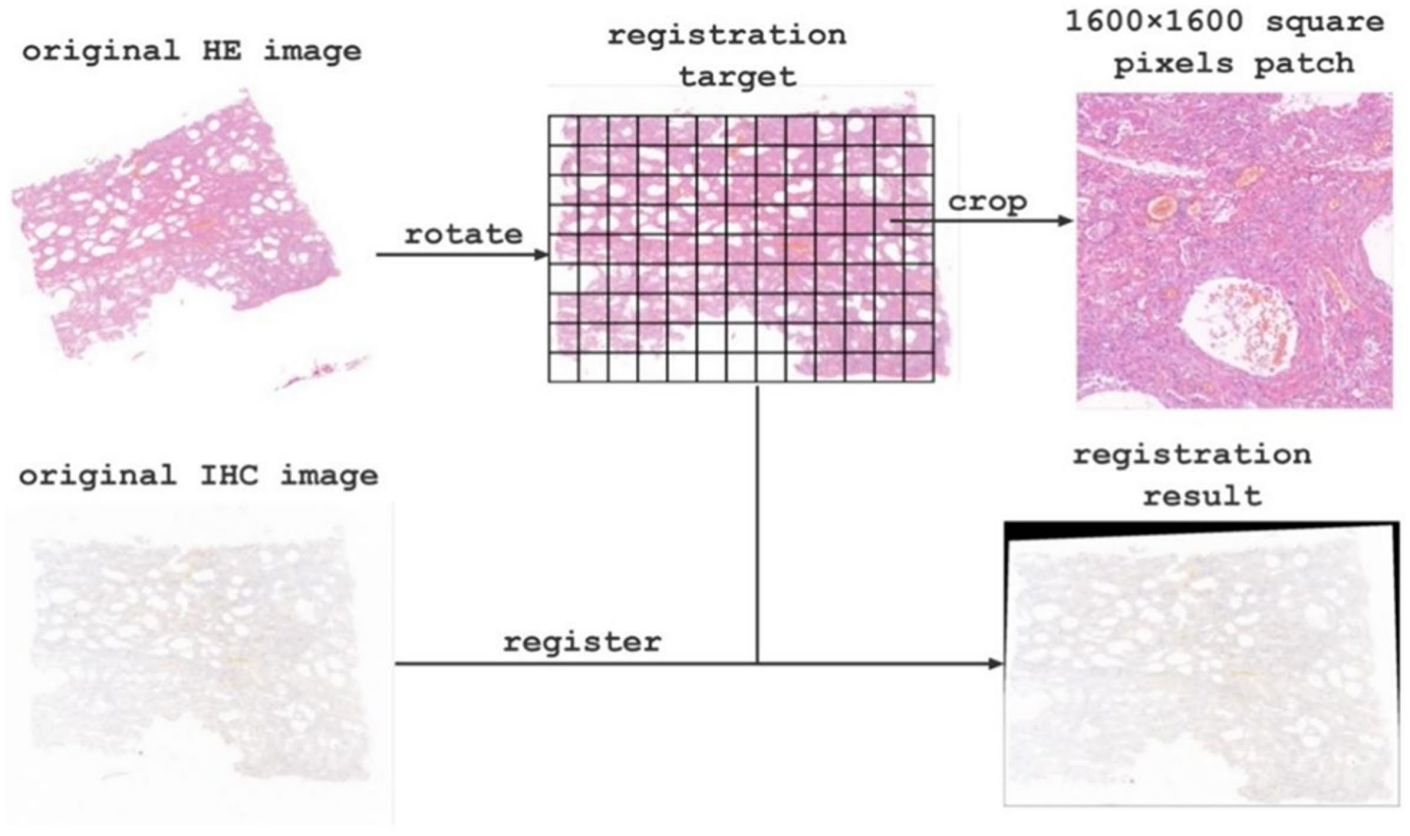
Image registration and cropping visualization.

The original images contained approximately 900 million pixels. After image registration and manual delineation to maximize the target area, the samples were cropped into 1600×1600 patches for subsequent model training and analysis (as shown in Figure 3 for the registration and segmentation results).

### 3.2. Classification experiments

#### 3.2.1. Evaluation metrics

We utilized the widely-used metrics: Precision, Recall and F1_score to evaluate the performance of fibrosis degree classification. Precision refers to the proportion of correctly predicted positive samples among all samples predicted as positive, as shown in Eq. (4). In Eqs. (4, 5) TP (True Positive) represents the number of positive samples correctly predicted as positive by the model, FP (False Positive) represents the number of negative samples incorrectly predicted as positive, and FN (False Negative) represents the number of positive samples incorrectly predicted as negative. Recall refers to the proportion of correctly predicted positive samples among all actual positive samples. F1_score is the harmonic mean of precision and recall as shown in Eq. (6), which comprehensively reflects the predictive ability and coverage of the model.


(4) $$\mathrm{Precision}=\frac{\mathrm{TP}}{\mathrm{TP}+\mathrm{FP}}\times \%100$$

(5) $$\mathrm{Recall}=\frac{\mathrm{TP}}{\mathrm{TP}+\mathrm{FN}}\times \%100$$

(6) $$F1\_\mathrm{score}=\frac{2\times \mathrm{Precision}\times \mathrm{Recall}}{\mathrm{Precision}+\mathrm{Recall}}\times \%100$$

#### 3.2.2. Results of classification

The results of pulmonary fibrosis degree classification are shown in Table 2.

**Table 2:** Results of pulmonary fibrosis degree classification.

Method	Precision	Recall	F1_Score
ResNet18	0.928	0.941	0.934
Transformer	0.936	0.925	0.929
ResNet+Transformer(w/ o SSL)	0.954	0.960	0.957
ResNet+Transformer(w/ SSL)	0.960	0.966	0.963

**Figure 4:**
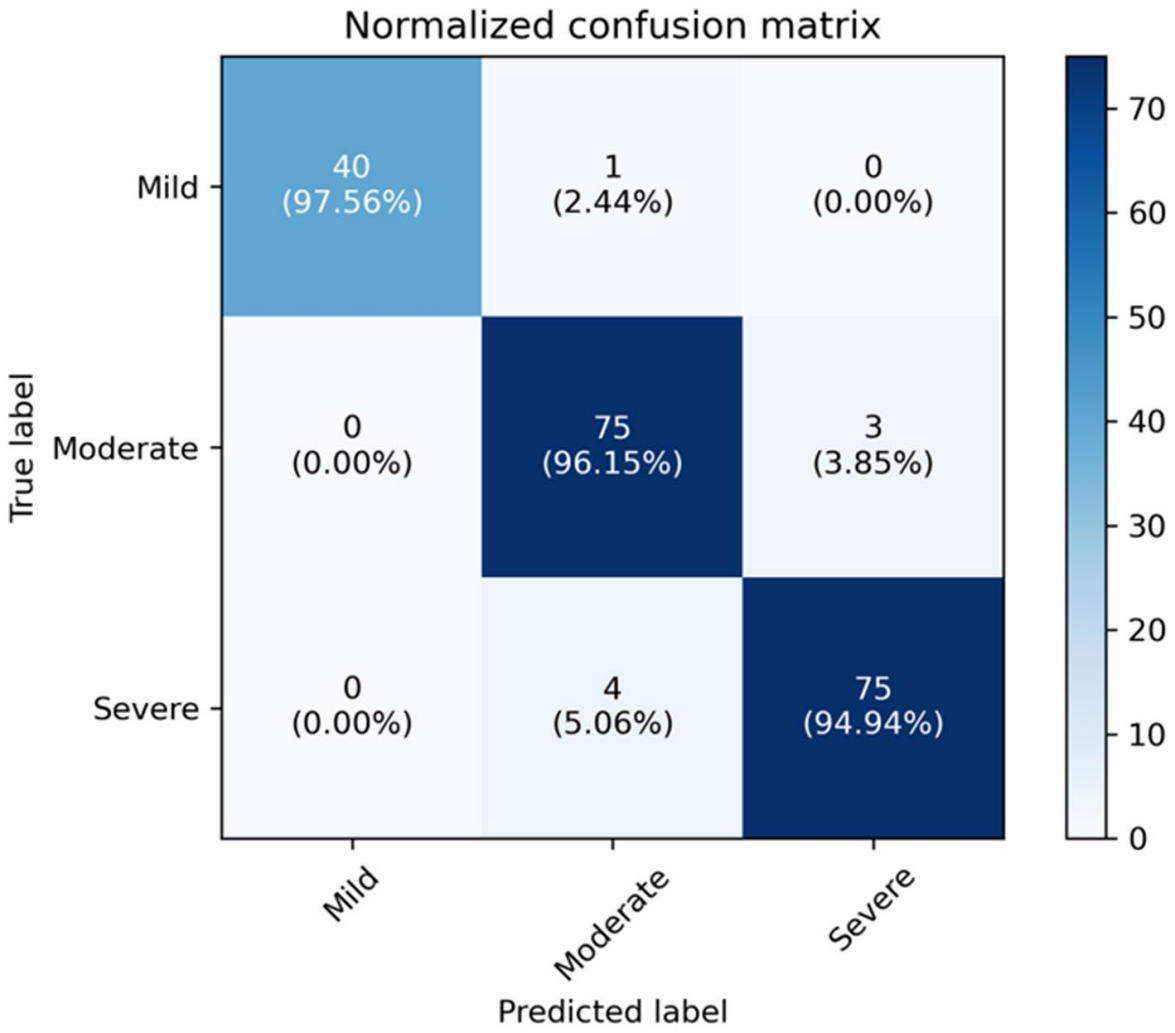
The normalized confusion matrix for the classification task.

We compare our final method, i.e., ResNet+Transformer (w/SSL) with several variants, including using only ResNet18 for classification, using only Transformer for classification, using ResNet+Transformer without semi-supervised learning (SSL). Experimental results suggest that utilizing ResNet as a feature extractor in combination with a Transformer network leads to a significant improvement in performance compared to using either ResNet or Transformer individually. Additionally, using semi-supervised learning can further enhance the model’s capability to focus on the target area and analyze complex samples. The normalized confusion matrix for the classification task is shown in Figure 4.

#### 3.2.3. Visualization of lung tissue fibrosis classification

**Figure 5:**
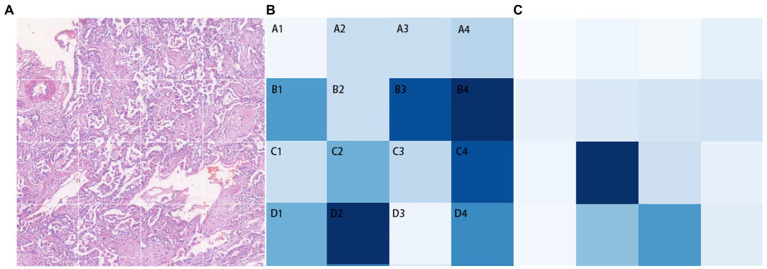
Classification task visualization. From left to right is the original input image **(A)**, the token map with SSL method **(B)** and the token map without SSL **(C)**.

In Transformer, the key component is the multi-head attention module. In each head, the correlation between every two tokens is calculated to obtain an attention map. We focus on those values involving the class token, which represent the degree of the influence of features from different patches on the classification decision. The attention maps in all heads (4 heads) of the multi-head attention module of each layer (10 layers) are extracted, then averaged and the elements related to the class token(calculated between a patch’ s token and the class token) are selected and adjusted to a two-dimensional matrix in order to match them with the corresponding locations in the original image for visualization. The shades of color are used to indicate the relative position of each vector to the class token in this two-dimensional space. Darker shades represent closer relative distances (i.e., higher correlation with the classification result), while lighter shades represent farther relative distances (i.e., lower correlation with the classification result; Figure 5). The B4 and D2 regions are the darkest in the image, indicating that the corresponding areas of pathological images have more prominent fibrosis features. As a comparison, the token map without SSL(as shown as Figure 5C), though it can also focus on some of the fibrotic areas, the areas of focus are incomplete and partially incorrect.

**Figure 6:**
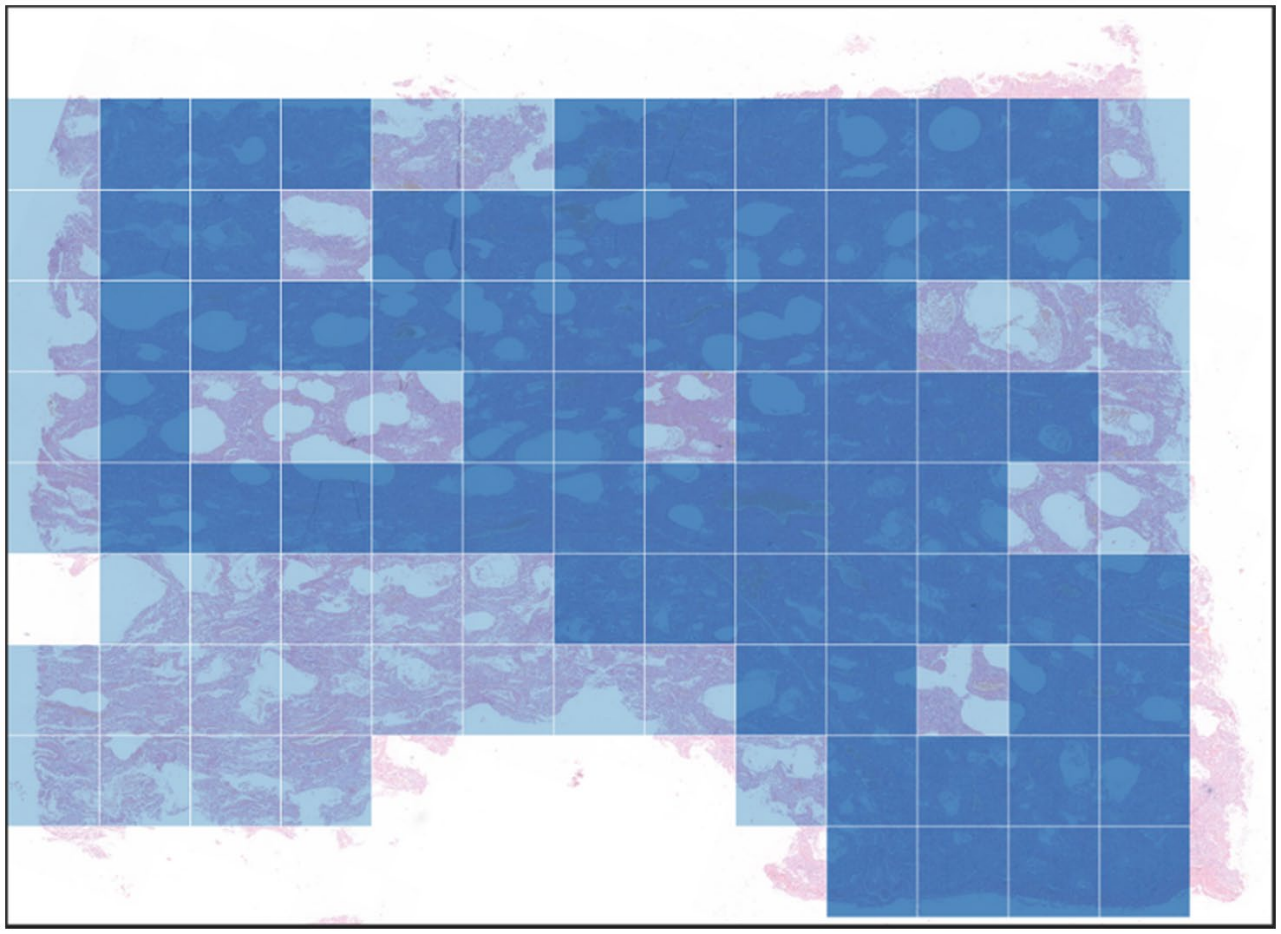
Global visualization map.

In the global visualization map (Figure 6), the deep blue region represents heavy fibrosis classification, the lighter region represents moderate fibrosis classification, and the colorless region is not included in the study due to insufficient tissue.

### 3.3. Results of cell counting and calibration

**Figure 7:**
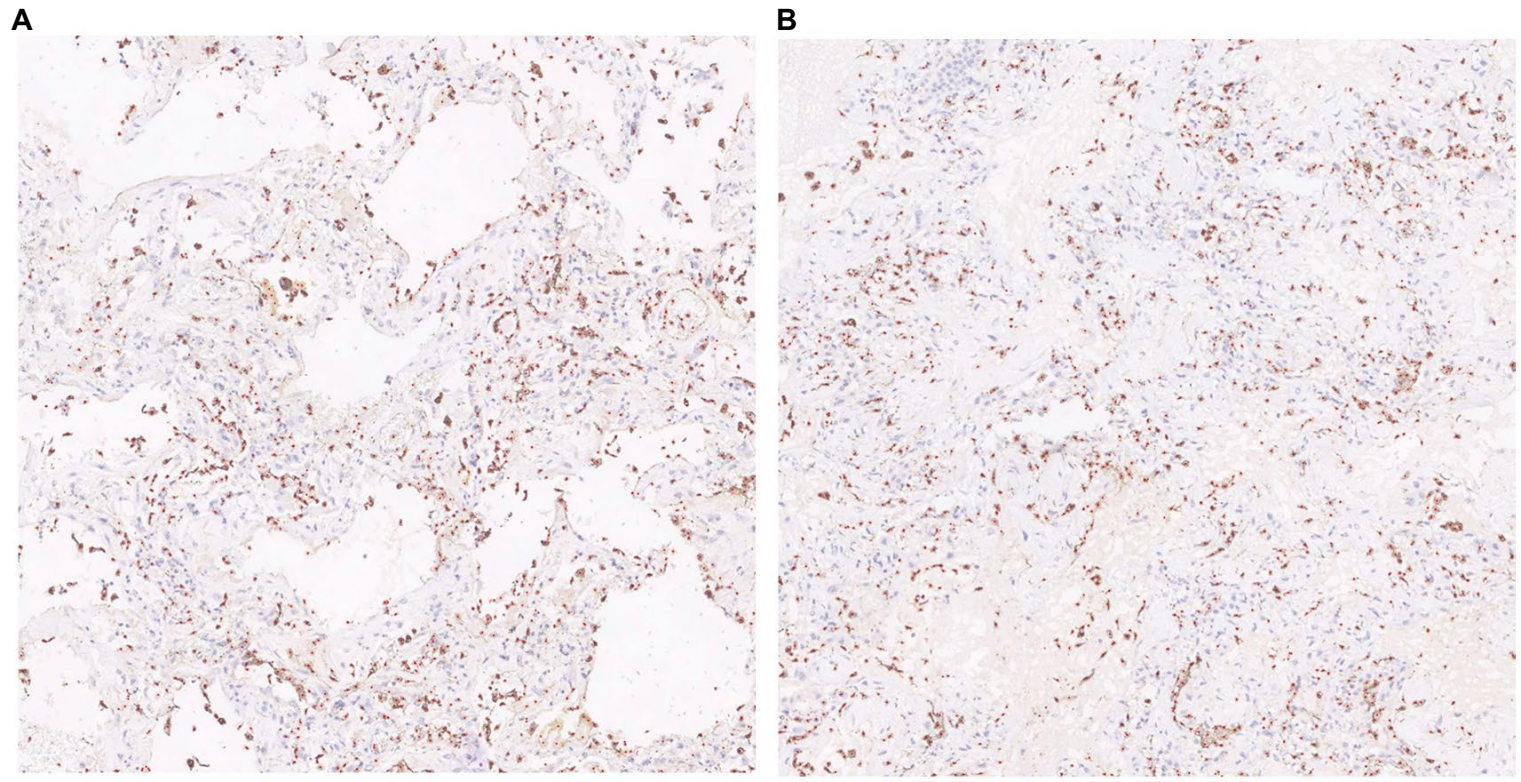
Visualization of cell prediction results (red dots are predicted cell locations). **A**:The patch contains some white regions; **B**:The patch only contains tissue.

The visualization results of the positive cell identified by our cell counting method in immunohistochemical staining images (Figure 7), in which Figure 7A contains some white regions, while 7B only has tissue. Direct counting and calibrated counting results of positive cells in Figure 7 are shown in Table 3.

**Table 3:** Cell count results of patch with white area (Figure 7A) and patch only contains tissues (Figure 7B).

Program	Figure 7A	Figure 7B
Before calibration	867.0	994.0
After calibration	851.0	859.0

The second and the third row correspond to the original counting results and the density results after the effective area calibration.

### 3.4. Results of image analysis of Covid-19 lung tissue sections

#### 3.4.1. Lung tissue image patch fibrosis classification

**Figure 8:**
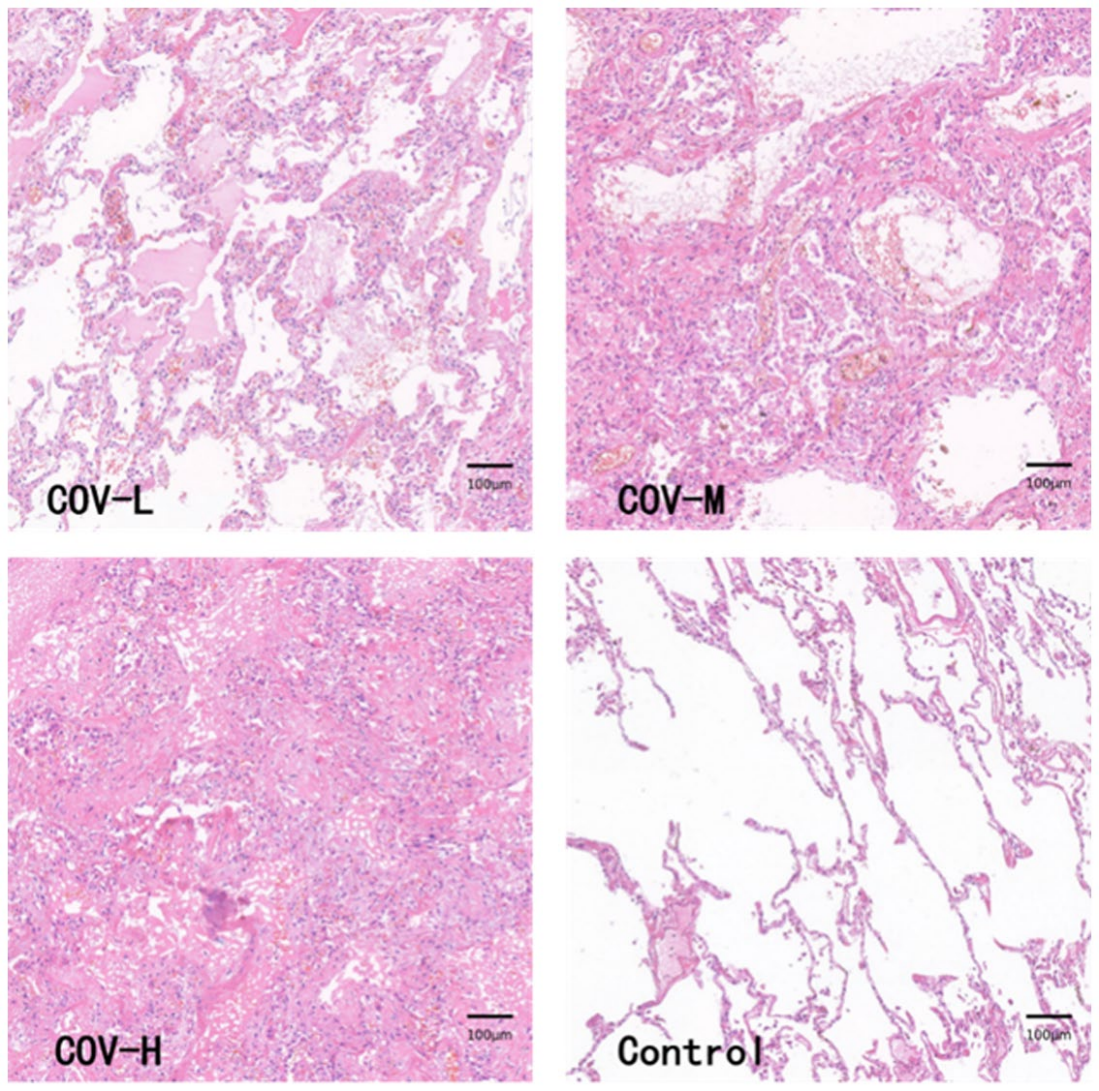
HE staining PATCH (1,600 × 1,600 pixels).

The HE-stained section images of the new coronal lung tissue were cut into 282 patches, and after the classification, the lung fibrosis light group (COV-L) 10 patches, the lung fibrosis moderate group (COV-M) 144 patches, the lung fibrosis heavy group (COV-H) 128 patches, and the normal lung tissue sections were segmented into 477 control groups (Control). The pathological images of lung tissues in each group are shown in Figure 8. Because the number of patches in the COVID-19 light group was too small, they were combined with COVID-19 moderate and analyzed as COVID-19 light-moderate group (COV-L/M) to reduce the error, reflecting the early stage of COVID-19 fibrosis.

#### 3.4.2. Morphological manifestations of immunohistochemical staining of macrophages in each group of COVID-19 and control group

**Figure 9:**
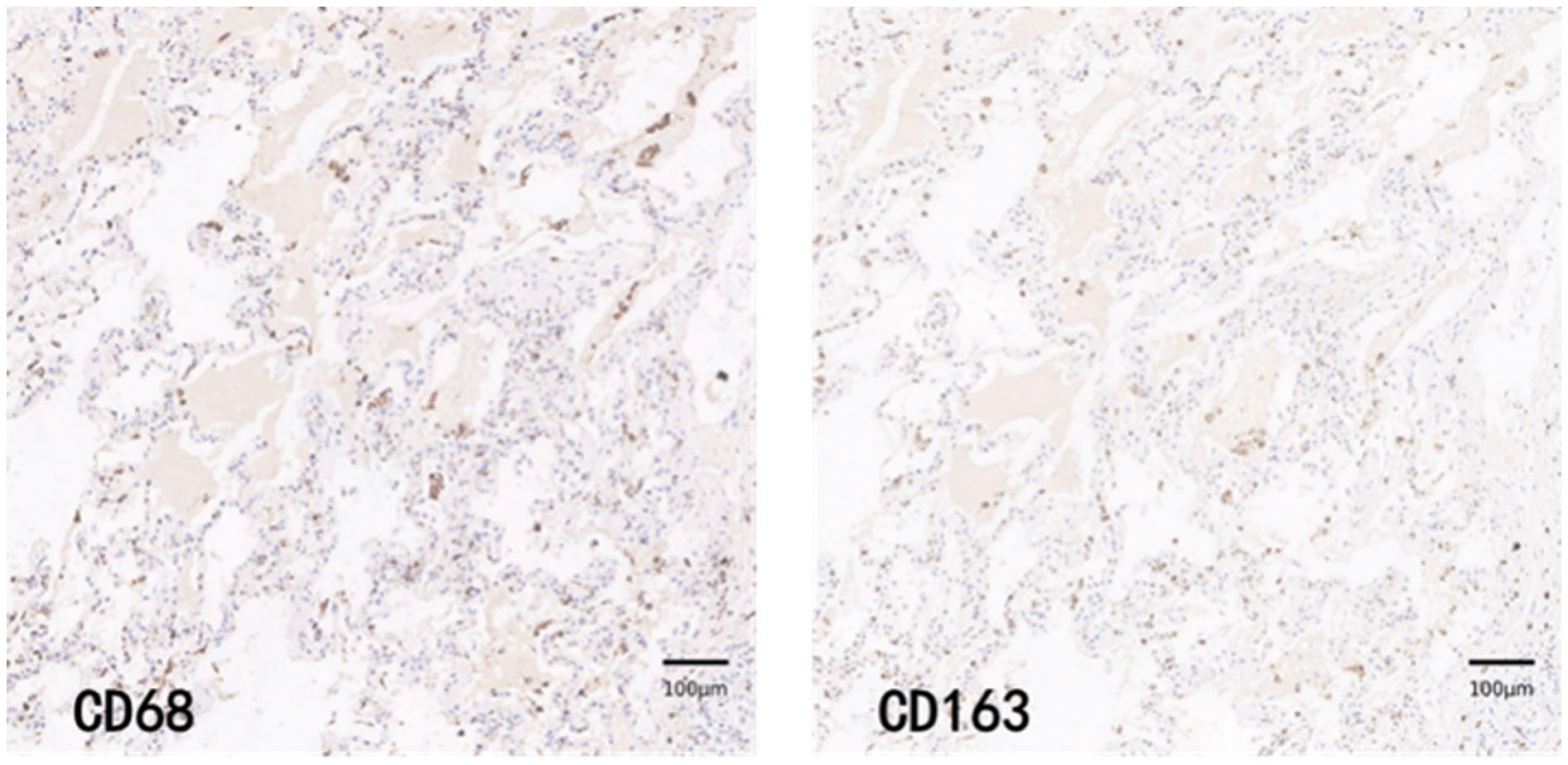
COV-L group immunohistochemical staining PATCH (1,600 × 1,600 pixels).

**Figure 10:**
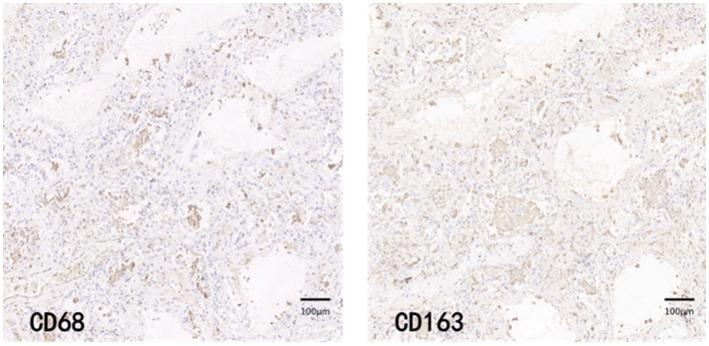
COV-M group immunohistochemical staining PATCH (1,600 × 1,600 pixels).

**Figure 11:**
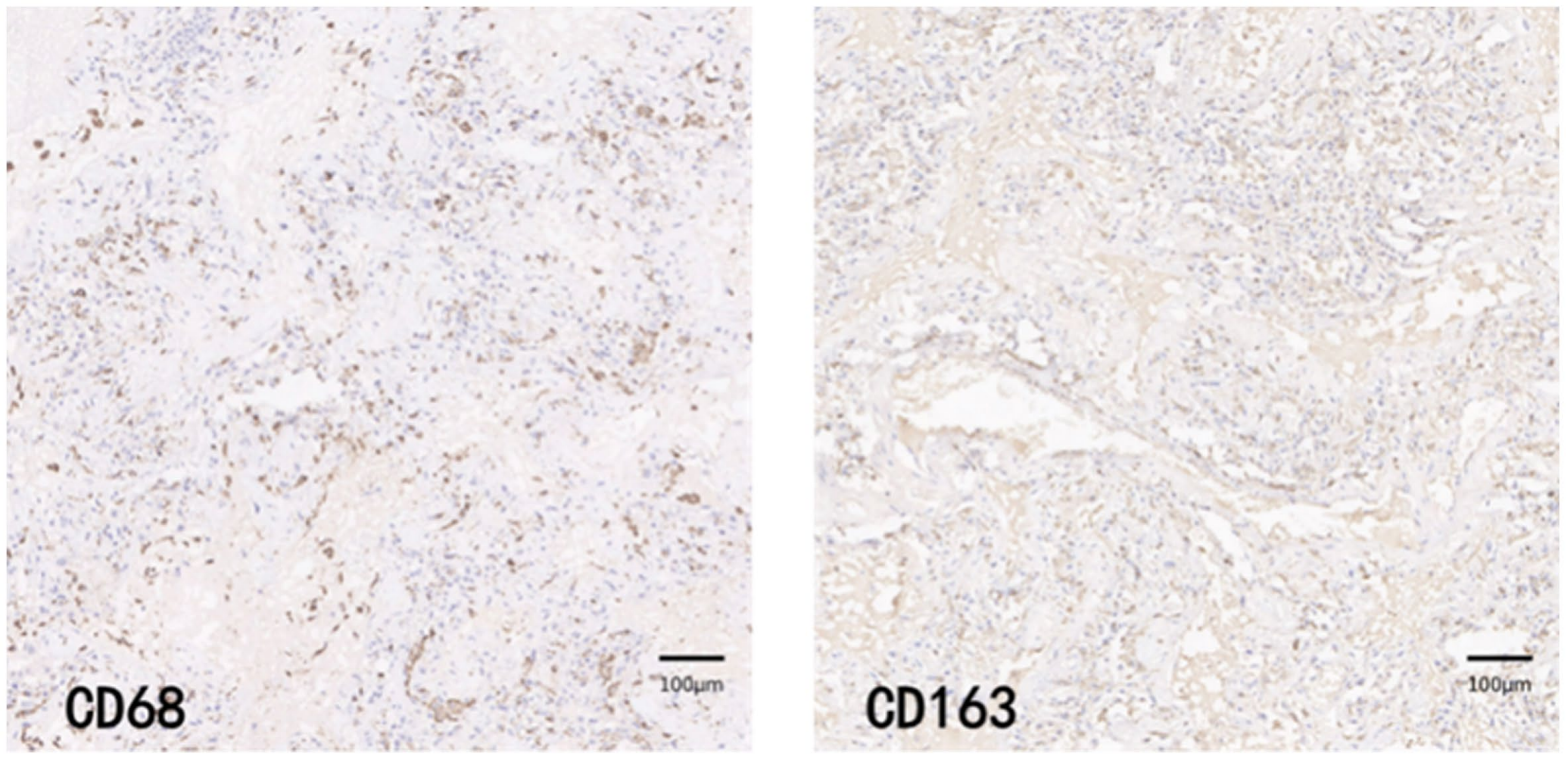
COV-H group immunohistochemical staining PATCH (1,600 × 1,600 pixels).

**Figure 12:**
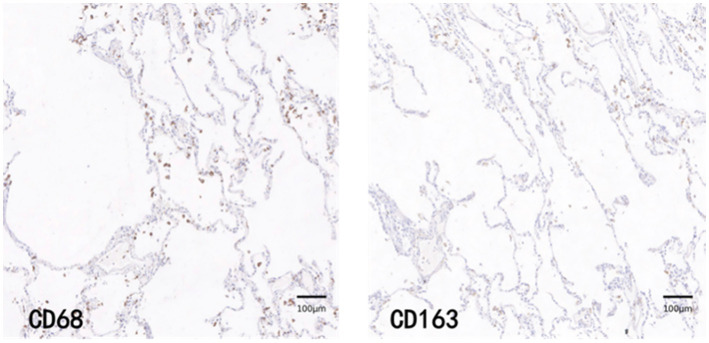
Control immunohistochemical staining PATCH (1,600 × 1,600 pixels).

CD68 and CD163 immunohistochemically positive cells were yellow-brown darkly stained cells in the sections. The common results of these four immunohistochemistry groups were characterized by a higher number of CD68 positive staining fine than CD163 positive staining cells in the field of view (e.g., Figures 9–12).

#### 3.4.3. Macrophage counts in COV-L/M Group, COV-H group and control group

The results of macrophage counts and M2/total macrophage ratios in the COV-L/M, COV-H and Control groups are shown in Table 4.

**Table 4:** Results of macrophage counting in COVID-19 group, paraquat group and Control group.

	CD68 (Total macrophages)	CD163 (M2-type macrophages)	M2/total macrophage ratio
COVID-19 light to moderate group	940.2 ± 289.8	792.2 ± 272.2	1.015 ± 0.7863
COVID-19 heavy group	982.0 ± 355.1	1,020 ± 299.0	1.367 ± 1.070
Control group	440.7 ± 214.2	168.9 ± 135.9	0.3681 ± 0.2383

Values are mean ± standard deviation.

**Figure 13:**
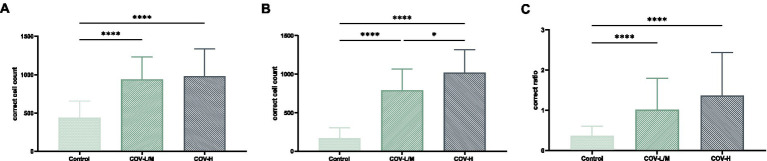
Comparative histogram of each macrophage count in each COVID-19 group and Control group **(A)** total macrophages, **(B)** M2-type macrophages, [**(C)** M2/total macrophage ratio; *p < 0.05, ****p < 0.0001].

The total macrophage count and M2/total macrophage ratio were significantly higher in the COV-L/M and COV-H groups compared with the Control group, but there was no statistical difference between the COV-L/M and COV-H groups (e.g., Figures 13A,C). The M2-type macrophages were elevated in the COV-H and COV-L/M groups compared with the Control group, and the elevation in the COV-H group compared with the COV-L /M group were more significantly elevated (e.g., Figure 13B).

## 4. Discussion

Image registration can be understood as the consistency of two or more images in spatial coordinates or visual perception. In pathological research, different staining methods are used to display different structures or components of the same tissue. In this case, it is necessary to align the images of different staining methods to the same coordinate system. The AI-based image analysis method proposed in this paper solves the problem of image registration for histological sections. By registering the HE-stained images with the immunohistochemical CD68 and CD163-stained images, the differences caused by changes in orientation for each section are eliminated, allowing HE staining results to be combined seamlessly with immunohistochemical results. Therefore, the image registration method proposed in this paper can unify information from different staining methods and lay the foundation for subsequent correlation analysis of research indicators.

In the process of building the classification model, we compared several state-of-the-art models in the literature with ResNet, which served as the baseline. By utilizing various combinations of data augmentation, we were able to achieve the best performance, as shown in Table 2. In this task, since image features are highly abstract, ResNet is better equipped to capture the complex features in the images. On the other hand, the Transformer pays more attention to local features and spatial relationships. However, because only one convolutional layer is used for feature extraction, the Transformer is not as effective as ResNet in this regard. After using the pre-trained ResNet model as a feature extractor, the model was able to extract higher-dimensional features, leading to significant improvements in model accuracy, recall, and F1 score. Furthermore, after augmenting the unlabeled samples with semi-supervised learning, the model’s performance was further enhanced. We observed that, with the use of SSL, our model provides more accurate and reasonable feature representations for different target regions, as evidenced by the visualized token map results.

From an application perspective, our proposed method is not only applicable to lung fibrosis classification, but also has broad potential applications in other medical imaging tasks or industrial image classification that require similar features. However, the current model also has certain limitations. For example, since the task is limited by the number of light cases, we supplemented some control cases into the light group, making it difficult for the current model to distinguish between the light and control groups. Patches with limited useful information were removed during annotation and were not included in the training set. In addition, the differences in semi-supervised learning algorithms may also affect the performance of the model. Currently, the two mainstream directions are to explore more suitable pseudo label strategies and to start with consistency learning to explore better data augmentation methods. In future, researcher could continue to optimize the model from points above.

By combining cell counting with effective area calibration, the counting algorithm presented in this paper can to some extent mitigate the influence of large blood vessel and bronchial lumens on lung parenchyma area, providing a more accurate reflection of the number of target cells in the lung parenchyma. Compared with fibrosis classification annotation, manually annotating cells in each image requires much more effort than classification, which is a major burden for researchers. Therefore, designing algorithms that can learn features from a small amount of annotation is a future research direction.

This paper is the first to apply artificial intelligence-based image analysis methods to systematically analyze lung tissue samples from COVID-19 patients. The samples analyzed in this paper showed predominantly moderate to heavy levels of lung fibrosis, with only a small proportion showing light fibrosis, which may be attributed to the fact that the samples were obtained from COVID-19 patients with underlying conditions. HE staining revealed that the light fibrosis group had a higher incidence of exudative lesions in the pulmonary alveolar spaces, such as edema and inflammatory cells, and the pulmonary alveolar septa were slightly thickened without the formation of fibrotic nodules. In the moderate fibrosis group, fibrotic nodules appeared but did not exceed 50% of the field of view. Almost all cases in the heavy fibrosis group showed fibrotic masses with only a small amount of free space.

Through immunohistochemical staining and statistical analysis, it was found that compared to the Control group, the COVID-19 group had significantly elevated levels of total macrophages and M2-type macrophages. Furthermore, there was a positive correlation between the level of M2-type macrophages and the degree of fibrosis, with the level of M2-type macrophages being higher in the heavy fibrosis group than in the light to moderate fibrosis groups. These findings suggest that COVID-19 leads to the accumulation of macrophages and an increase in M2-type polarization, which is associated with the severity of pulmonary fibrosis. The M2/total macrophage ratio was significantly increased in all COVID-19 groups compared to the Control group, but there was no difference between the different fibrosis groups, which may be due to the increase in both total macrophages and M2-type macrophages during disease progression. These results indicate that both the total number of macrophages and the M2-type polarization phenomenon are significant factors in COVID-19-induced pulmonary fibrosis.

In summary, macrophages play an important role in the process of COVID-19-induced pulmonary fibrosis, with the number of M2-type macrophages positively correlated with the degree of fibrosis. The specific molecular mechanism by which M2-type macrophage polarization leads to pulmonary fibrosis warrants further exploration.

## 5. Conclusion

Pulmonary fibrosis is not only a common complication of microbial infection of the lung but a serious threat to human health, and its pathogenesis and associated targets need to be further explored. In this paper, an artificially intelligent image analysis method is developed to align, slice, and discriminate the degree of fibrosis in microscopic images of lung tissue. The application of the artificial intelligence image analysis method constructed in this paper enables a standardized, precise, and staged study of pulmonary fibrosis, providing a more standardized method and more accurate data for the correlation of the degree of pulmonary fibrosis and aiding in the treatment and prevention of pulmonary fibrosis.

Furthermore, in this paper, we also applied this newly developed artificial intelligence image analysis method to explore the mechanism of Post-COVID-19 pulmonary fibrosis. We found that the accumulation of macrophages is a common pathological manifestation of Post-COVID-19-induced pulmonary fibrosis, in which M2-type macrophages play a major role. In the future, the related signaling molecules before and after the polarization of M2-type macrophages can be further explored to identify the key targets of Post-COVID-19-induced pulmonary fibrosis.

## Data availability statement

The raw data supporting the conclusions of this article will be made available by the authors, without undue reservation.

## Ethics statement

The studies involving human participants were reviewed and approved by Ethics Committee of Tongji Medical College, Huazhong University of Science and Technology. The patients/participants provided their written informed consent to participate in this study.

## Author contributions

QL and XY were responsible for the construction of the overall study design and the overall revision of the paper. YC was responsible for the construction of the cell counting algorithm model and calibration method. LL built the algorithm model for image alignment and automatic classification. HH was responsible for providing pathology image data and performing data analysis. KL assisted in the development of the algorithm model. MJ, XZ, and SL were involved in the annotation and analysis of the pathology image data. All authors contributed to the article and approved the submitted version.

## Funding

This work was supported by the Natural Science Foundation of Hubei Province (2021CFA053), the National Natural Science Foundation of China (62061160490) and the Applied Fundamental Research of Wuhan (2020010601012167).

## Conflict of interest

The authors declare that the research was conducted in the absence of any commercial or financial relationships that could be construed as a potential conflict of interest.

## Publisher’s note

All claims expressed in this article are solely those of the authors and do not necessarily represent those of their affiliated organizations, or those of the publisher, the editors and the reviewers. Any product that may be evaluated in this article, or claim that may be made by its manufacturer, is not guaranteed or endorsed by the publisher.
